# Fixation of an Osteochondral Fragment with Autologous Bone Picks in the Knee – A Case Report

**DOI:** 10.1055/s-0044-1787550

**Published:** 2024-08-01

**Authors:** Elemar da Silva Resch, Sandrey da Rosa Machry, Caio César Zottis, Fernando Knoll Barros

**Affiliations:** 1 Ortopedia e Traumatologia, Hospital Universitário de Santa Maria, Santa Maria, RS, Brasil; 2 Ortopedia e Traumatologia, Hospital Independência, Porto Alegre, RS, Brasil

**Keywords:** bone and bones, knee fractures, osteochondritis

## Abstract

Osteochondral injuries in the knee are uncommon in the immature skeleton and are usually related to sporting activities.

Fixation is required depending on the size and location of the fragment. The standard technique is open reduction and internal fixation with metal screws, which are removed in a second procedure after consolidation.

As an alternative to reduce risk and morbidity, fixation of the osteochondral fragment may use autologous bone picks.

This study reports the execution of this surgical technique on a 13-year-old patient who injured his right knee during a soccer match.

## Introduction

Articular cartilage is a connective tissue covering the bony surfaces of joints. It is avascular, highly hydrated, and has no neural components. 1 In joint trauma, injuries can be purely chondral or associated with bone fragments, configuring an osteochondral injury. 2 3

The standard technique for fixation is open reduction and internal fragment fixation with screws. However, these screws require removal in a second surgical procedure. Bioabsorbable synthetic material use is not widespread and can induce foreign body reactions. 4 As such, this study aims to report a technique for osteochondral fragment fixation in the knee using autologous bone sticks from tibial cortical bone.

## Case Report

The Research Ethics Committee of our institution approved this case report under the number (CAAE 68381222.0.0000.5304). The patient's guardian signed the informed consent form.

**Fig. 1:**
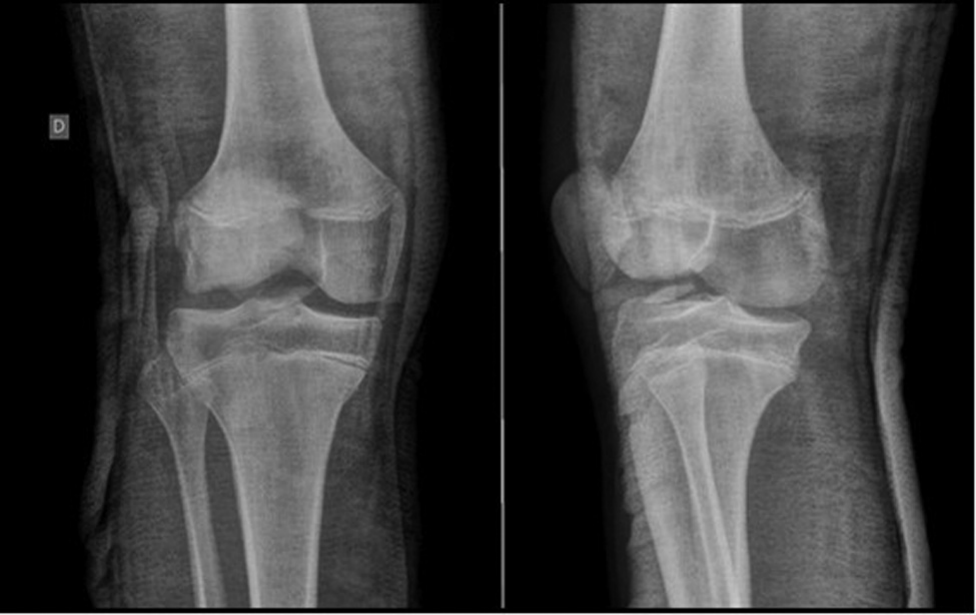
Initial X-ray.

A male, 13-year-old patient, previously healthy, arrived at the consultation with a history of trauma to the right knee, 2 days prior, resulting from a valgus stress mechanism during soccer practice. Since the event, the patient had moderate pain, inability to mobilize the knee, and 2 +/4+ effusion. The radiographic examination revealed a dislocated osteochondral fragment of the lateral condyle of the right knee measuring approximately 3 cm in its largest diameter ( Fig. 1 ).

Therefore, we opted for surgical treatment, i.e., open reduction and internal fixation with the cortical bone stick technique.

**Fig. 2:**
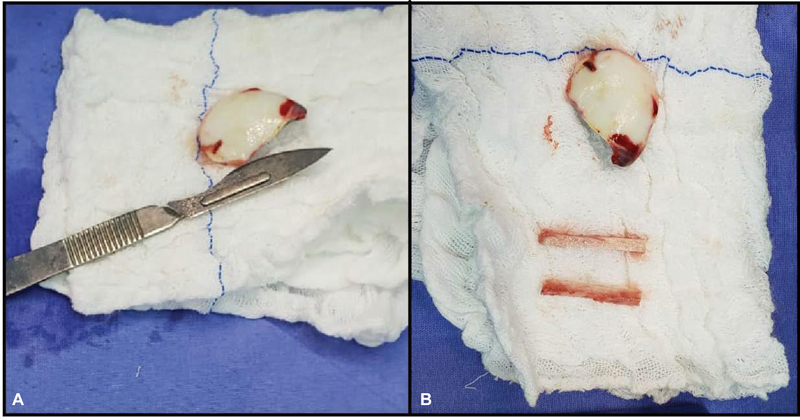
Osteochondral fragments.

The surgery occurred 14 days after initial care. We performed a longitudinal lateral parapatellar arthrotomy, measuring approximately 8 cm, on the patient's right knee. Under direct visualization, the anatomical site was cleaned and grafted, and the fragment measuring 35 mm × 27 mm was identified ( Fig. 2A ).

**Fig. 3:**
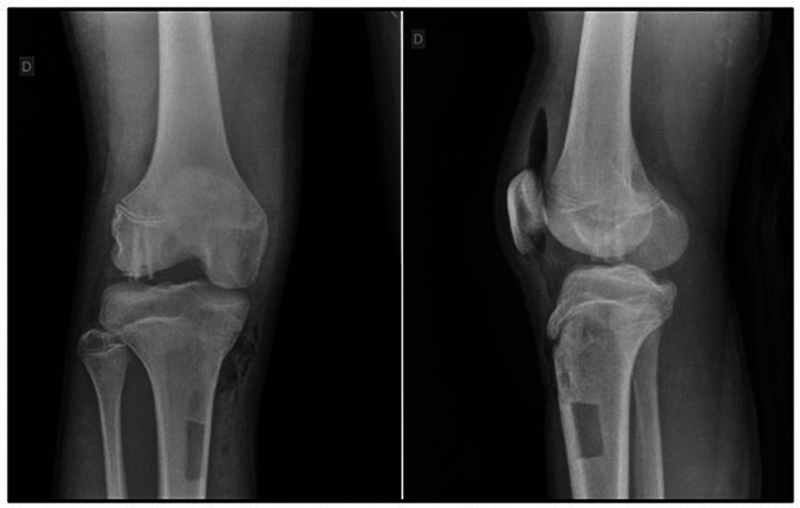
Post operative X-ray.

Next, we removed a cortical window from the middle diaphysis of the ipsilateral tibia to create bone sticks. To do so, we made a 4-cm longitudinal skin incision over the anteromedial proximal metaphysis of the right tibia. After elevating the periosteum, we opened a window measuring approximately 30 × 15 mm, longer in the longitudinal direction, with a saw to make bone sticks ( Fig. 2B and Fig. 3 ).

**Fig. 4:**
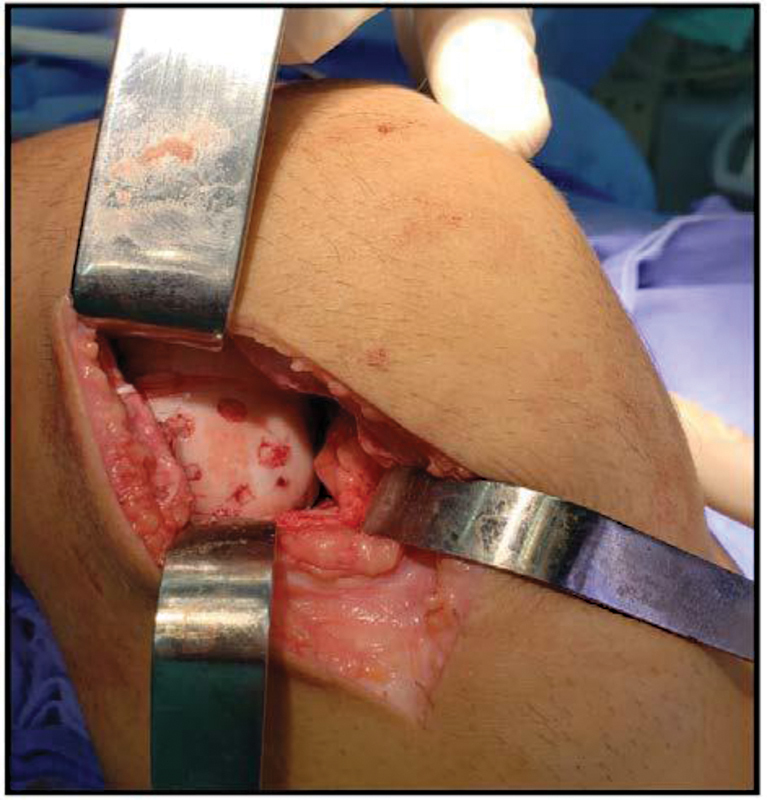
Surgery image.

We prepared 5 cortical bone sticks, each measuring approximately 3 mm in diameter and 30 mm in length. The provisional fixation of the osteochondral fragment employed 2.5-mm Kirschner wires. Each Kirschner wire was successively replaced by a bone stick. Subsequently, we lowered the sticks to enter 3 mm into the articular surface. It is worth highlighting that the fixation of the chondral fragment at the site of origin, with the five bone sticks, occurred through an angular insertion divergent to the surface ( Fig. 4 ).

**Fig. 5:**
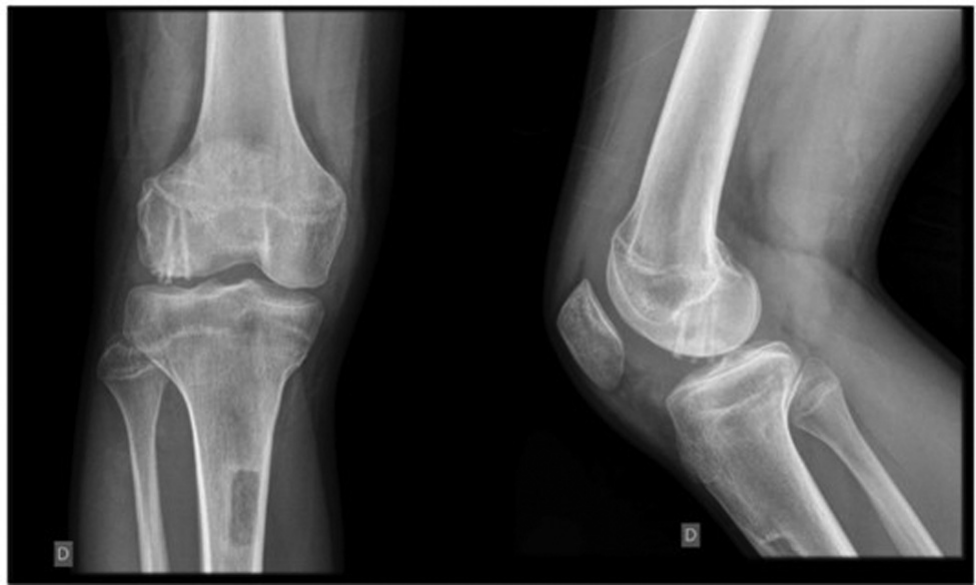
X-ray 4 months after the surgery.

At discharge, we instructed the patient to perform exercises for early limb movement with no support. Two weeks after surgery, he started motor physical therapy, walking with crutches and no support. We allowed partial loading at 2 months and full loading as tolerated at 4 months after surgery. The patient underwent periodical supplementary imaging exams confirming the good evolution and right knee recovery. At 4 months, the radiological examination demonstrated a proper bone consolidation of the fractured fragment ( Fig. 5 ).

**Fig. 6:**
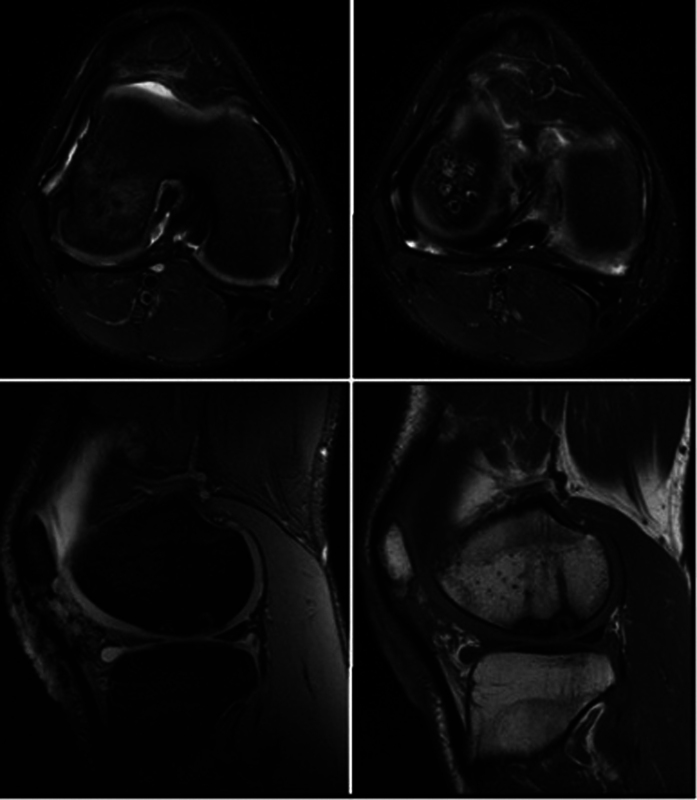
Magnetic resonance 1 year and 2 months after the surgery.

The patient resumed his regular sports and impact activities 1 year and 2 months after surgery. A follow-up magnetic resonance imaging (MRI) scan demonstrated the good evolution of joint reconstruction ( Fig. 6 ).

## Discussion

Osteochondral fractures of the lateral femoral condyle are infrequent. 3 Physical examination may reveal edema, movement limitation, and joint blockage. The investigation must begin with a plain radiography. Pure chondral injuries require other imaging tests, such as MRI and arthroscopy. 5

After diagnosing an osteochondral fracture, the anatomy of the articular surface needs restoration to avoid early osteoarthrosis. The most common fixation technique employs metal screws. Despite being efficient and widely used, the screws have to be removed in a second surgical procedure. To overcome this issue, bioabsorbable synthetic materials have emerged for fragment fixation. However, these materials are not widespread and can generate a foreign body reaction. 4

The fixation technique with bone sticks has already been used as a form of fixation in osteochondritis dissecans of the knee, and numerous studies have proven the good bone consolidation provided. 6 7 The sticks are made from cortical bone, usually from the tibia. The number of sticks used for fixation in osteochondritis dissecans is variable; some studies report an average of 3 to 4 sticks per lesion, 8 while others describe 4 to 6. 7

In the present case report, we selected five rectangular bone sticks. The incompatibility between the circular hole and the square stick ensures a firmer fixation. 8 Bone sticks can be described as autologous bone grafts. Thus, for fracture consolidation, they have osteoconductive and osteoinductive properties. 9

The advantage of using bone sticks to fix osteochondral fragments in the knee is allowing fracture fixation in a single surgery with no risk of a foreign body reaction. Despite the short follow-up period and the lack of histological follow-up of the lesion, imaging tests, and the patient's complete physical rehabilitation demonstrate that fixation of the osteochondral fragment of the knee with bone sticks is an effective technique.
