# Supplementary material for: Fixation of an Osteochondral Fragment with Autologous Bone Picks in the Knee – A Case Report
Source: Rev Bras Ortop (Sao Paulo). 2024 Aug 1;59(Suppl 2):e145–8. doi: 10.1055/s-0044-1787550 (PMC11679627; doi:10.1055/s-0044-1787550)
Supplement: Related texts

# Research article: Fixação de fragmento osteocondral com palitos ósseos autólogos em joelho *–* Um relato de caso

**Keywords:** fraturas do joelho, osso e ossos, osteocondrite

## Abstract

Lesões osteocondrais no joelho são incomuns no esqueleto imaturo e costumam estar relacionadas com atividades esportivas.

De acordo com o tamanho e a localização do fragmento, a fixação se faz necessária. A técnica padrão é realizada através da redução aberta e fixação interna com parafusos metálicos, que precisam retirados em um segundo procedimento, após consolidação.

Como alternativa, visando a redução de risco e morbidade, existe a fixação do fragmento osteocondral com palitos ósseos autólogos.

O presente trabalho relata a execução dessa técnica cirúrgica em um paciente de 13 anos que lesionou o joelho direito durante uma partida de futebol.

## Introdução

A cartilagem articular é um tecido conectivo que recobre as superfícies ósseas das articulações, sendo descrita como avascular, aneural e altamente hidratada. 1 Nos traumas articulares, as lesões podem ser puramente condrais ou associadas com fragmentos ósseos, configurando uma lesão osteocondral. 2 3

A técnica padrão para fixação é realizada através da redução aberta e fixação interna do fragmento com parafusos. No entanto, estes precisam ser removidos em um segundo tempo cirúrgico. O uso de materiais sintéticos bioabsorvíveis ainda não são amplamente utilizados e podem induzir reações de corpo estranho. 4 Nesse contexto, este trabalho tem como objetivo relatar uma técnica de fixação de fragmento osteocondral no joelho, na qual foram confeccionados palitos ósseos autólogos, a partir de osso cortical tibial, para fixação.

## Relato de caso

Este relato de caso foi aprovado no Comitê de Ética em Pesquisa da nossa instituição sob o número (CAAE- 68381222.0.0000.5304), e o formulário de consentimento informado foi fornecido e assinado pelo responsável do paciente.

Um paciente do sexo masculino, com 13 anos de idade, previamente hígido, chegou à consulta com história de trauma no joelho direito, com 2 dias de evolução, por mecanismo de stress em valgo durante prática de futebol. Desde o evento, apresentava dor moderada, com incapacidade de mobilizar, associado a derrame 2 +/4 + . No exame radiográfico, evidenciou-se fragmento osteocondral do côndilo lateral do joelho direito deslocado, com aproximadamente 3 cm em seu maior diâmetro ( Fig. 1 ).

**Fig. 1:** Raio-x inicial.

Desta forma optou-se por tratamento cirúrgico, por meio de redução aberta e fixação interna através da técnica de palitos ósseos corticais.

A cirurgia foi executada após 14 dias do atendimento inicial. Realizou-se artrotomia parapatelar lateral longitudinal, de cerca de 8 cm, no joelho direito do paciente. Através de visualização direta, executou-se a limpeza e cruentização do sítio anatômico e identificou-se o fragmento que apresentava dimensões de 35 mm × 27 mm ( Fig. 2A ).

**Fig. 2:** Fragmentos osteocondrais.

Posteriormente, foi retirada uma janela da cortical da diáfise média da tíbia ipsilateral, para a confecção dos palitos ósseos. Para isso, foi realizada uma incisão cutânea longitudinal de 4 cm sobre a metáfise proximal ântero-medial da tíbia direita. Após a elevação do periósteo, foi aberta uma janela de aproximadamente 30 × 15 mm, de maior comprimento no sentido longitudinal, com uma serra, para a confecção de palitos ósseos ( Fig. 2B e Fig. 3 ).

**Fig. 3:** Raio-x pós-operatório.

Foram confeccionados 5 palitos ósseos corticais, cada um com cerca de 3 mm de diâmetro e 30 mm de comprimento. O fragmento osteocondral foi fixado provisoriamente com fios de Kirschner de 2,5 mm. Cada fio de Kirschner foi sucessivamente substituído por um palito ósseo. Posteriormente, os palitos foram rebaixados, adentrando em 3 mm na superfície articular. Além disso, é importante salientar que a fixação do fragmento condral no sítio de origem, com os 5 palitos ósseos, foi através de inserção angular divergente em relação à superfície ( Fig. 4 ).

**Fig. 4:** Imagem da cirurgia.

Na alta, o paciente foi instruído a realizar exercícios para movimentação precoce do membro, sem apoio. Com 2 semanas de pós-operatório iniciou fisioterapia motora, deambulando com muletas, sem apoio. Aos 2 meses iniciou carga parcial, e aos 4 meses foi liberada carga total conforme tolerância. Periodicamente, o paciente realizou exames complementares de imagem, os quais confirmaram a boa evolução e recuperação do joelho direito. Aos 4 meses de evolução, o exame radiológico demonstrou um bom processo de consolidação óssea do fragmento fraturado ( Fig. 5 ).

**Fig. 5:** Raio-x 4 meses após a cirurgia.

Com 1 ano e 2 meses do pós-operatório retornou às atividades esportivas e de impacto normalmente. O exame de ressonância magnética (RM) de controle demonstra a boa evolução da reconstrução articular ( Fig. 6 ).

**Fig. 6:** Ressonância magnética 1 ano e 2 meses após a cirurgia.

## Discussão

As fraturas osteocondrais do côndilo femoral lateral são patologias infrequentes. 3 Ao exame físico, podem apresentar edema, limitação de movimento e bloqueio articular. A investigação é iniciada pelo exame de radiografia simples. Quando se trata de lesões condrais puras, outros exames de imagem se fazem necessários, como a RM e artroscopia. 5

Uma vez diagnósticada fratura osteocondral, a anatomia da superfície articular precisa ser restaurada a fim de evitar uma osteoartrose precoce. A técnica mais utilizada para fixação utiliza parafusos metálicos. Apesar de ser eficiente e amplamente utilizada, os parafusos precisam ser removidos em um segundo tempo cirúrgico. Com o objetivo de contornar esse problema, surgiram materiais sintéticos bioabsorvíveis para fixação fragmentar; porém, estes ainda não são amplamente utilizados e podem gerar reação de corpo estranho. 4

A técnica de fixação com palitos ósseos já tem sido usada como forma de fixação na osteocondrite dissecante de joelho, e inúmeros trabalhos comprovam a boa consolidação óssea proporcionada. 6 7 Os palitos são confeccionados a partir de cortical óssea, geralmente da tíbia. O número utilizado para fixação na osteocondrite dissecante é variável, com alguns trabalhos demonstrando uma média de 3 a 4 palitos por lesão, 8 outros 4 a 6. 7

No presente relato de caso, optou-se por fazer uso de 5 palitos ósseos, de formato retangular. A incompatibilidade entre o orifício circular e o palito quadrado garante uma fixação mais firme. 8 Os palitos ósseos podem ser descritos como enxertos ósseos autólogos. Assim, na consolidação de fraturas, apresentam propriedades osteocondutivas e osteoindutivas. 9

A utilização de palitos ósseos para fixação de fragmentos osteocondrais no joelho tem a vantagem de permitir a fixação da fratura em apenas um tempo cirúrgico, sem risco de reação de corpo estranho. Apesar do curto período de seguimento e da ausência de acompanhamento histológico da lesão, os exames de imagem, bem como a total reabilitação física do paciente, demonstram que a fixação de fragmento osteocondral de joelho por meio de palitos ósseos é uma técnica eficaz.
